# Hemophagocytic Lymphohistiocytosis secondary to Rickettsial infection: A case report

**DOI:** 10.1002/ccr3.6730

**Published:** 2022-12-12

**Authors:** Shriya Sharma, Aakriti Adhikari, Nirmal Ghimire, Gaurab Mainali, Sumit Kumar Yadav, Prasamsa Pudasaini, Shashank Neupane

**Affiliations:** ^1^ Nepalese Army Institute of Health Sciences Kathmandu Nepal; ^2^ Department of Internal Medicine Nepal Police Hospital Kathmandu Nepal; ^3^ Department of Internal Medicine Shree Birendra Hospital Kathmandu Nepal

**Keywords:** hemophagocytic lymphohistiocytosis, hemophagocytosis, *Orientia tsutsugamushi*, scrub typhus

## Abstract

Hemophagocytic Lymphohistiocytosis (HLH) is a rare life‐threatening condition characterized by widespread activation of the immune system leading to tissue damage all over the body. It is divided into primary HLH due to inborn error in lymphocytes, T cells, and macrophages and secondary HLH which is mostly due to infections, systemic connective tissue diseases, and lymphoid malignancies. Here, we report a 34‐year‐old man with a history of high‐grade fever, chills, and rigor, eschar, splenomegaly with the laboratory findings of thrombocytopenia, hypochromic RBCs with anisocytosis and basophilic stippling, elevated transaminases, and a positive Weil Felix test along with positive PCR results for Orientia tsutsugamushi and the presence of IgG and IgM antibodies. A detailed workup was done to rule out other etiology for fever. Diagnosis of HLH secondary to Rickettsia infection was made with a thorough history, clinical evaluation, and a variety of investigations. The patient was treated with Doxycycline, Ciprofloxacin, Etoposide, and Dexamethasone but unfortunately, the patient died during treatment due to multiorgan failure. Patients with scrub typhus typically respond well to therapy; therefore, early detection and antibiotic treatment can help avoid serious complications. Scrub typhus with the hemophagocytic syndrome can result in DIC and multiorgan failure. Despite its rarity, scrub typhus may be lethal; as a result, practitioners must be aware of the necessity of detecting and treating suspected cases as soon as possible. We learned that a systematic diagnostic approach, use of diagnostic criteria, and prompt treatment are very crucial in this disease.

## Graphical abstract

We discussed about a systematic diagnostic approach, use of diagnostic criteria, and prompt treatment in Hemophagocytic Lympho‐Histiocytosis secondary to Rickettsial infection which is crucial in this disease.

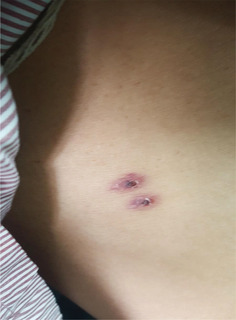

## 1 INTRODUCTION

Hemophagocytic lymphohistiocytosis is an aggressive and life‐threatening condition with excessive activation of cytotoxic T lymphocytes, natural killer (NK) cells, and macrophages resulting in hypercytokinemia and immune‐mediated damage to multiple organ systems. Primary (driven via underlying genetic mutations) or secondary (driven by a malignant, infectious, or autoimmune stimulus without an identifiable underlying genetic trigger).^1^ However, this classification does not represent correctly the complexity of the genetic defects in HLH, in which biallelic severe mutations lead to earlier onset HLH, and mild mutations lead to later onset HLH. The mortality rate of adult HLH ranges from 26.5% to 74.8% according to studies and étiologies.^1^

Progress has been made regarding the pathophysiology of HLH over the past decade. However, the diagnosis of HLH remains with many dilemmas because of the heterogeneous nature of the disease.^2^

The guidelines for hemophagocytic lymphohistiocytosis describe the various symptoms that can be considered for a clinical diagnosis. Presence of immunosuppression, Fever, Organomegaly, Elevation in triglyceride levels, Elevation of ferritin levels, Elevations of aspartate aminotransferase, Presence of cytopenias, Hemophagocytosis on bone marrow aspirate. HLH may present with intravascular lymphoma, T‐cell histiocyte‐rich large B‐cell lymphoma, and angioimmunoblastic T‐cell lymphoma. So bone marrow biopsy is important to rule out hemophagocytosis and to assess the malignancy associated with HLH.^3^

Excessive monocyte activation in HLH could be caused by high amounts of activating cytokines. High levels of interferon (IFN), soluble interleukin‐2 receptor, tumor necrosis factor (TNF), interleukin‐1, and interleukin‐6 have been found, implying that T‐helper cells are elaborating activating cytokines or that poorly regulated or inappropriate Th1 responses to intracellular pathogens are to blame. Although the details of immunological protective mechanisms against rickettsial infections, such as cytokine activation, are unknown, macrophages and T cells are thought to play a key role in rickettsial infection protection and the emergence of HLH. HLH is caused by a variety of viruses, including CMV, Epstein–Barr virus (EBV), and human herpesvirus‐6, as well as collagen‐vascular disorders and malignancies, including T‐cell lymphomas.^4^

## 2 CASE REPORT

**FIGURE 1:**
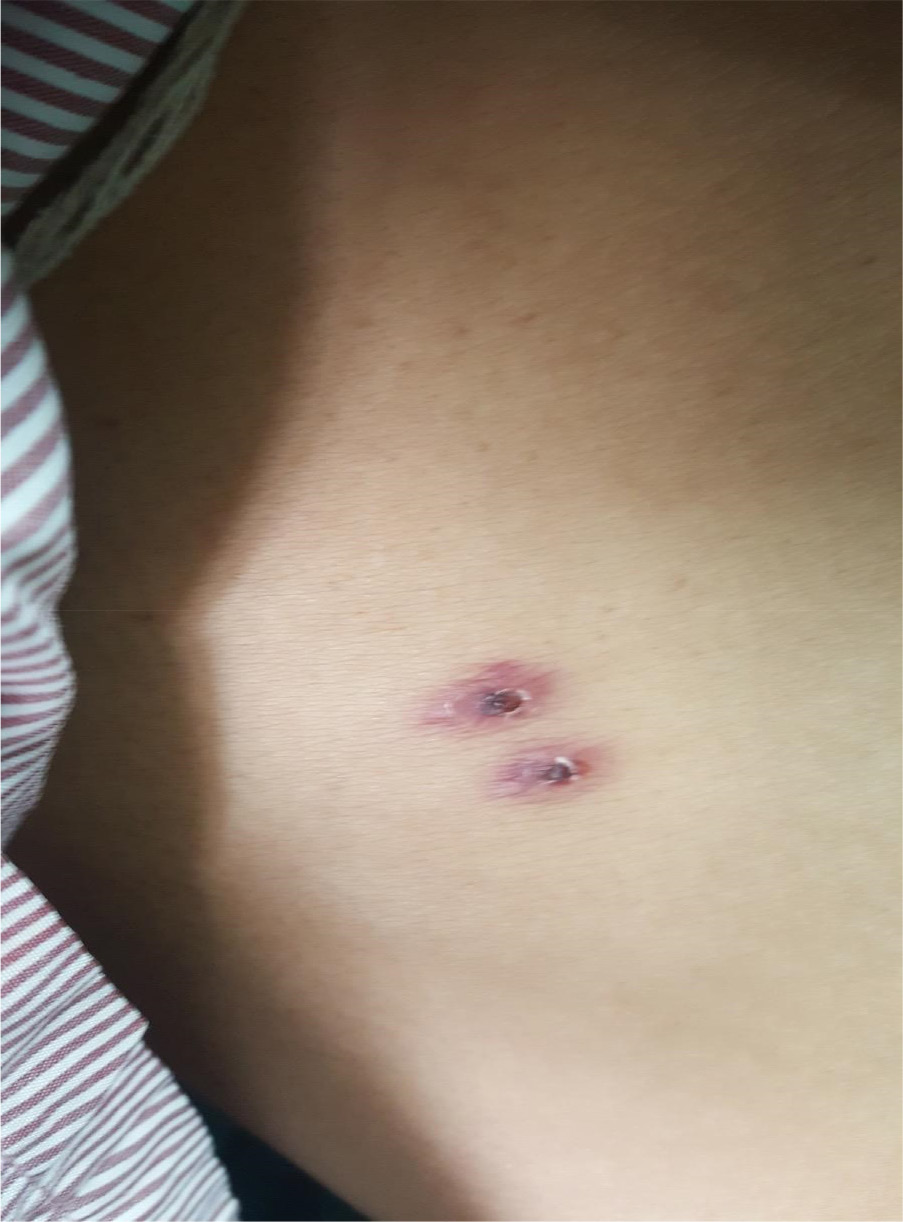
Typical eschar seen in scrub typhus

A 34‐year‐old male patient presented to the emergency department with a history of high‐grade intermittent fever, associated with chills and rigor along with a frontal headache with no rash or bleeding diathesis. There is no history of cough, sore throat, pain abdomen, burning micturition, earache/discharge, nausea, or vomiting. There was the presence of two similar dark scab‐like lesions with erythematous bases around 1 cm in diameter on the abdomen (Figure 1). He was later admitted for further evaluation and a definitive diagnosis. In view of thrombocytopenia (platelet count – 97,600/cmm, raised transaminases (AST – 164 IU/dL and ALT – 156 IU/L), LDH (2780 IU/L), CRP 147.4 mg/dL, (0.3–1.0 mg/dL) with no other localizing signs, and a positive Weil Felix Test, he was treated as a case of Rickettsia fever with Doxycycline and Ciprofloxacin tablets. He was hemodynamically stable but continued to have fever(101–102 F) with chills and rigors which was intermittent. The patient was receiving supportive care, including IV fluids and paracetamol for fever. The fever began to fade during his stay, but it was predicted to last for around a month, according to the infectious diseases specialist.

He was evaluated in the line of infective, inflammatory, autoimmune, and neoplastic possibilities for the fever. To rule out infectious processes as a cause for fever, PPD tests and malarial blood smear were done for tuberculosis and malaria which were negative. Similarly, workup was done to rule out EBV, CMV, Hepatitis A, Hepatitis B, Hepatitis C, HIV 1 and 2, Dengue fever, Salmonella, and Brucella infections which were negative.

**FIGURE 2:**
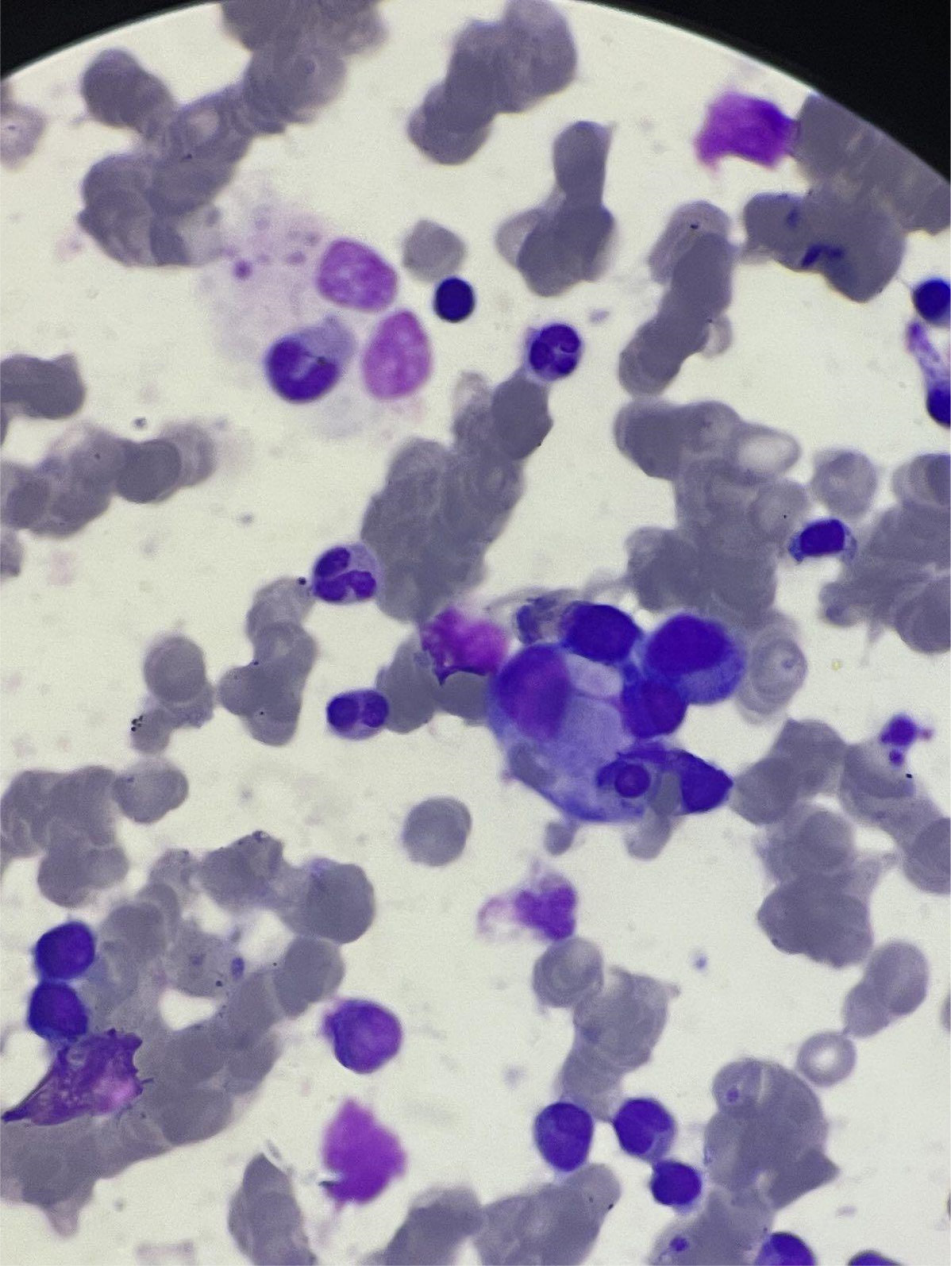
Increased number of histiocytes and emperipolesis seen in bone marrow biopsy

Then, a peripheral blood smear was done where hypochromic RBC with anisocytosis and basophilic stippling along with‐it Leukopenia and giant platelets were seen. For further evaluation, Bone marrow biopsy was done which showed Dys‐erythropoietic features with erythrophagocytic cells (Figure 2). NK cell activity test was not done because it was not available in our institution. In the line of investigation to rule out malignancy, a CT scan of the chest, abdomen, and pelvis was done. An abdomen CT scan showed a spleen with an upper limit size measuring 14 cm. Whole body FDG PET/CT was done which showed mildly enlarged spleen measuring up to 13 cm in length, demonstrating mild diffuse hypermetabolism of liver activity. The remaining low‐attenuation splenic lesions were not appreciated with certainty on the current unenhanced CT, without focal abnormal FDG uptake. However, a few small upper abdominal lymph nodes are seen demonstrating faint FDG uptake just above background liver activity, including at the gastrohepatic ligament, periportal, and right superior diaphragmatic regions, such as a 12 × 8 mm gastrohepatic ligament lymph node. There is normal FDG activity throughout the remainder of the abdomen and pelvis.

After this extensive workup, a diagnosis of hemophagocytic lymphohistiocytosis secondary to Rickettsial infection was made with the following findings:Triglyceride: 267 mg/dL, Ferritin: >2000 mcg/dl (30–300 ng/mL), Fibrinogen: 98 mg/dL, Bone marrow aspiration and biopsy: Hyperactive macrophages with erythrophagocytosis, LDH: 2780 U/L, Bi‐cytopenia: Hb:10.9 g/dL TLC: 3800/microliter. Hscore of the patient was 200.

He was started on dexamethasone 10 mg/m^2^ after which the patient started getting better. Further treatment was dexamethasone 10 mg/m^2^ once daily for 2 weeks followed by tapering to 4–5 mg/m^2^, Etoposide 150 mg/m^2^ twice weekly for 6 weeks followed by once weekly and reassessment accordingly. The patient then developed abdominal pain in the right and left upper quadrant along with petechial spots in the abdomen. He also developed a high‐grade fever of 103 F along with features suggestive of septic shock. He was shifted to ICU due to his deteriorating condition and deranged hematological panel. During his stay in ICU, the patient died because of MODS.

## 3 DISCUSSION

O. tsutsugamushi being a mite‐borne bacterium poses the risk of a serious disease called Scrub Typhus. Rodents function as animal reservoirs for O. tsutsugamushi; however, the microorganism also can be maintained among mite colonies through transovarial transmission.^5^ Studies show scrub typhus is an evolving public health problem with numerous outbreaks since 2015 in Nepal. Scrub typhus is a neglected tropical disease and is one of the important causes of undifferentiated treatable fever in Asia.^6^

Scrub typhus is associated with eschar, which is a pathognomonic lesion. It is the first lesion that appears after being bitten by a chigger (Leptotrombidium mite). Because eschar is where O. tsutsugamushi is multiplying and a huge number of organisms are found there, it has been demonstrated to be a superior sample for PCR test than blood.^7^ Scrub typhus is defined by small vessel vasculitis, which affects the lungs, heart, brain, and kidneys in particular. Scrub typhus has non‐specific clinical signs, and patients frequently report to the physician with a generalized fever of uncertain etiology. Severe symptoms of MOF, ARDS, shock, and DIC, on the contrary, may develop.^8^

Primary HLH occurs due to hereditary immune conditions, while on the contrary secondary HLH occurs in settings such as infection, malignancy, autoimmune disease, post‐allogeneic hematopoietic stem cell transplantation, and drug hypersensitivity.^5^ Interconnection between HLH and infection is vital as both familial and sporadic cases are commonly provoked by infectious diseases. HLH imitates infectious diseases concealing the identification of a causative agent leading to serious health problems.^9^ Recent studies have shown that adult HLH is associated more with pro‐inflammatory genetic and functional defects, while HLH is associated more with cytotoxicity defects.^10^

Patients with HLH die because of bleeding in visceral organs, opportunistic infection secondary to neutropenia, or multiple organ failure within 2 months which accounts for more than 10% of cases.^5^

With an increasing number of cases documented in the last 10 years, HLH is a potentially serious consequence of scrub typhus. However, the majority of instances recorded thus far have been single cases or case series with a limited sample size. As a result, the clinical symptoms and prognosis of individuals with HLH linked with scrub typhus are mostly unknown.^2^ In the present case, the patient had a high‐grade intermittent fever with eschar which was not responding to broad‐spectrum antibiotics. During the initial work, the patient was diagnosed with a case of Scrub typhus not responding to Doxycycline and Ciprofloxacin. For this reason, an extensive workup was carried out to rule out other etiologies of fever which failed to establish the cause. Bi‐cytopenia and Hyperactive macrophages with erythrophagocytosis in bone marrow biopsy suggested the possibility of HLH. Similarly, in line with HLH, biochemical parameters met the criteria for diagnosis of HLH. The patient initially responded well to the treatment but eventually, his condition deteriorated due to multiorgan failure leading to death. This patient succumbed to his disease, despite treatment with steroids and etoposide, which is the standard therapy in adult HLH.^11^ Indeed, one of the main difficulties in treating infection‐associated HLH is that etoposide may make it difficult for patients to recover from infection. JAK inhibition, particularly ruxolitinib, is becoming more commonly used in an HLH, and this option should be mentioned as an emerging therapy in infection‐associated HLH.^12^

## 4 CONCLUSION

Hemophagocytic lymphohistiocytosis (HLH) is a rare life‐threatening condition. Scrub typhus with hemophagocytic syndrome can result in DIC and multiorgan failure. Despite its rarity, scrub typhus may be lethal; as a result, practitioners must be aware of the necessity of detecting and treating suspected cases as soon as possible.

## AUTHOR CONTRIBUTIONS

**Aakriti Adhikari:** Supervision. **Nirmal Ghimire:** Project administration. **Gaurab Mainali:** Writing – review and editing. **Sumit Kumar Yadav:** Conceptualization; resources. **Prasamsa Pudasaini:** Methodology. **Shashank Neupane:** Validation.

## CONFLICT OF INTEREST

The authors declare that they have no competing interest.

## CONSENT

The authors confirm that the patient has provided written informed consent to the submission of this case report, in accordance with the journal's patient consent policy.

## Data Availability

Data available on request from the authors. The data that support the findings of this study are available from the corresponding author upon reasonable request.
